# Development and characterization of an immunochromatographic test for the rapid diagnosis of *Talaromyces (Penicillium) marneffei*

**DOI:** 10.1371/journal.pone.0195596

**Published:** 2018-04-11

**Authors:** Kritsada Pruksaphon, Akarin Intaramat, Kavi Ratanabanangkoon, Joshua D. Nosanchuk, Nongnuch Vanittanakom, Sirida Youngchim

**Affiliations:** 1 Department of Microbiology, Faculty of Medicine, Chiang Mai University, Chiang Mai, Thailand; 2 Laboratory of Immunology, Chulabhorn Research Institute, Bangkok, Thailand; 3 Chulabhorn Graduate Institute, Bangkok, Thailand; 4 Departments of Medicine (Infectious Diseases) and Microbiology/Immunology, Albert Einstein College of Medicine, Bronx, NY, United States of America; University of Minnesota, UNITED STATES

## Abstract

*Talaromyces* (*Penicillium*) *marneffei* is a thermally dimorphic fungus that can cause opportunistic systemic mycoses in patients infected with the human immunodeficiency virus (HIV). It has also been reported among patients with other causes of immunodeficiency, such as systemic lupus erythematosus, cancer, organ transplanted patients receiving immunosuppressive drug and adult onset immunodeficiency syndromes. Recent studies indicate that the clinical manifestations, laboratory findings and treatment strategies of talaromycosis (penicilliosis) marneffei are different between patients with and without HIV infection. Therefore early and accurate diagnosis of talaromycosis marneffei is crucial to the proper management and treatment. Since current diagnostic methods are currently inadequate, the aim of this study was to develop an immunochromatographic test (ICT) for the detection of *T*. *marneffei* yeast antigens in urine samples. The highly *T*. *marneffei-*specific monoclonal antibody 4D1 (MAb 4D1) conjugated with gold colloid at pH 6.5 was used as signal generator. The nitrocellulose membrane was lined with *T*. *marneffei* cytoplasmic yeast antigen (TM CYA) to serve as the test line, and rabbit anti-mouse IgG was the control line. Subjecting the assembled test strip to urine samples containing *T*. *marneffei* antigen produced a visible result within 20 minutes. The sensitivity limit of the assay was 3.125μg/ml of TM CYA. The ICT was used to test urine samples from 66 patients with blood culture confirmed talaromycosis marneffei, 42 patients with other fungal or bacterial infections, and 70 normal healthy individuals from endemic area of *T*. *marneffei*. The test exhibited sensitivity, specificity and accuracy of 87.87%, 100% and 95.5%, respectively. This rapid, user-friendly test holds great promise for the serodiagnosis of *T*. *marneffei* infection.

## Introduction

*Talaromyces marneffei* (previously named *Penicillium marneffei*) is classified as an important emerging opportunistic fungal infection. It is the most prevalent systemic mycotic infection in patients infected with human immunodeficiency virus (HIV). *T*. *marneffei* is endemic in tropical Asia including Thailand, northeastern India, southern China, Hong Kong, Vietnam and Taiwan [1–5]. Disseminated infection with *T*. *marneffei* is most often found in patients with secondary immunodeficiency syndromes, especially in patients with AIDS, where this mycoses is the third most common AIDS-defining opportunistic infections in tropical Asia, after tuberculosis and cryptococcosis [6–8]. Due to improved treatments of HIV infection and enhanced public health efforts, the incidence rate of HIV-associated *T*. *marneffei* infection has been significantly declining [8]. However, *T*. *marneffei* infection has concomitantly been increasingly recognized among patients with non–HIV associated immunodeficiency syndromes, such as systemic lupus erythematosus (SLE), cancer, organ transplanted patients receiving immunosuppressive drug and adult onset immunodeficiency syndromes [9–12]. In addition, *T*. *marneffei* infection was also found in non-HIV-infected hematology patients treated with novel targeted therapies, including anti-CD20 monoclonal antibodies and kinase inhibitors [13,14].

Notably, recent studies have indicated that the clinical manifestations, laboratory findings and treatment strategies of *T*. *marneffei* infection are different between patients with and without HIV infection [11]. The diagnosis of *T*. *marneffei* infection is difficult because its clinical manifestations may mimic tuberculosis, melioidosis, pneumocystosis, leishmaniasis, histoplasmosis and other AIDS-related opportunistic infections [15,16]. Indeed, preliminary diagnosis of *T*. *marneffei* infection is often made on the basis of microscopic identification of fission yeasts in macrophages or histiocytes from clinical specimens [16–18]. However, this methodology is subjected to limitation given that microscopically *T*. *marneffei* is similar to *Histoplasma capsulatum*, *Leishmania donovani*, and *Pneumocystis carinii* [19]. Hence, microbiological culture is the gold standard method for diagnosis of talaromycosis marneffei. However, this method requires prolonged incubation time (7–10 days), frequently resulting in the delay of appropriate antifungal therapy. In addition, the sensitivity of fungal culture from blood can be low (76.7%) in HIV-positive patients whilst only 47.1% in HIV-negative patients [11]. A number of other diagnostic methods have been developed, including serologic diagnostic method. Serological approaches have shown that HIV-infected patients with *T*. *marneffei* infection have lower levels of *T*. *marneffei* specific antibody and higher levels of *T*. *marneffei* antigen compared with HIV-negative *T*. *marneffei* infected patients [9]. Serodiagnosis for the detection of *T*. *marneffei* antigen in urine specimen has become an alternative technique for diagnosis. A combination of dot blot ELISA and a latex agglutination assay utilizing rabbit polyclonal antibody generated against killed whole-fission-form arthroconidia of *T*. *marneffei* were able to detect *T*. *marneffei* antigen in urine with 94.6% (35/37) and 100% (37/37) of sensitivity and specificity, respectively [15,20]. However, these approaches have not been applied in clinical practice. Tests utilizing monoclonal antibodies (MAbs) against the 76-kDa and 62-kDa of exoantigen derived from *T*. *marneffei* mycelial culture filtrate have low specificity [16,21], which is consistent with prior findings of significant cross reactivities between the antigens of *T*. *marneffei* and of other common pathogenic fungi including *Aspergillus fumigatus*, *Candida albicans*, *Cryptococcus neoformans*, *H*. *capsulatum*, *Coccidioides immitis*, *Paracoccidioides brasiliensis* and *Blastomyces dermatitidis* [6, 22–24].

Rafferty et al. [25] generated a highly specific monoclonal antibody, termed MAb 4D1, against *T*. *marneffei* cytoplasmic yeast antigen (TM CYA). MAb 4D1 (a mouse IgG1) is reactive against a 50–180 kDa of N–linked glycosylated mannoprotein present in the yeast phase of *T*. *marneffei*. In addition, the MAb 4D1 not only demonstrated phase specificity, but also demonstrated no cross-reactivity against antigens from other thermally dimorphic and other common pathogenic fungi including *H*. *capsulatum*, *P*. *brasiliensis*, *B*. *dermatitidis*, *Sporothrix schenckii*, *C*. *neoformans*, *C*. *albicans*, *A*. *fumigatus*, *A*. *flavus*, *Trichophyton tonsurans* and *Microsporum canis* [26]. Recently, we developed a novel inhibition enzyme linked immunosorbent assay (Inh-ELISA) using MAb 4D1 to quantify antigenic levels of *T*. *marneffei* from patient sera [27]. This assay specifically detected antigenemia in all 45 patients with culture-confirmed *T*. *marneffei* infection, with a mean antigen concentration of 4.32 μg/ml. However, the Inh-ELISA was laborious and time consuming.

Rapid lateral flow immunochromatographic test (ICT) systems have been developed for the serodiagnosis of many global infectious diseases such as malaria, AIDS, syphilis and viral hepatitis [28]. These assays usually consist of single use disposable strips that are simple, user-friendly, and rapid, and they also provide a low limit of detection together with high diagnostic sensitivity and specificity [29]. ICTs have recently been extensively utilized for systemic mycoses. For example, a sandwich format ICT based on two MAbs specific to the capsular polysaccharide glucuronoxylomannan of *C*. *neoformans* (CrAg) is widely used for the diagnosis of cryptococcosis [30]. More recently, a multi-host specific ICT based on the use of recombinant Proteins A/G for the detection of human and animal antibodies specific to the aquatic microorganism *Pythium insidiosum* has been developed for the serodiagnosis of the infection [31].

We report here the development of a novel lateral-flow immunochromatographic test (ICT) for the detection of *T*. *marneffei* antigen in urine using the *T*. *marneffei* yeast phase specific MAb 4D1. The diagnostic performance of the ICT was evaluated in comparison to that of hemoculture. The results indicate that this *T*. *marneffei* ICT has the potential to facilitate simple, rapid, and accurate diagnosis of diseases due to *T*. *marneffei* in HIV infected patients as well as other immunocompromised patients.

## Materials and methods

### Preparation of *T*. *marneffei* cytoplasmic yeast antigen (TM CYA) and the specific MAb 4D1

The preparation of TM CYA was performed as described by Jeavons et al. [32]. Briefly, *T*. *marneffei* ATCC 200051, at a concentration 5x10^6^ conidia/ml, was cultured in 150 ml of brain-heart infusion broth (BHI: Difco™) and incubated on a shaking incubator at 150 rpm at 37°C for 5 to 7 days. Thereafter, thimerosal (Sigma) was added to the final concentration of 0.02% (v/v) and incubated at room temperature overnight before harvesting. The yeast cells were harvested by centrifugation at 4500 rpm, 4°C for 10 min and then broken with 0.5-mm glass Ballotini beads (BioSpec, Inc.) in a homogenizer (BioSpec, Bartlesville, USA). Subsequently, the insoluble yeast cell remnant was removed by centrifugation at 10,000 rpm for 20 min at 4°C and the CYA was collected from the supernatant. The protein purity and immunoreactivity of the prepared TM CYA were examined by dye binding method, sodium dodecyl sulfate polyacrylamide gel electrophoresis (SDS-PAGE), immunoblotting and indirect ELISA [32]. The cytoplasmic antigens of other common pathogenic fungi were prepared following the same procedures. The MAb 4D1 hybridoma clone was cultured in serum free medium (Gibco™), and purified by HiTrap™ column protein G affinity chromatography (GE™ Healthcare) as described [33]. The specificity of MAb 4D1 was studied using indirect ELISA and western blotting [27].

### Determination of mannoprotein secretion to the *T*. *marneffei* culture supernatant

To determination of immunogenic mannoprotein target of MAb 4D1 in culture supernatant, the culture supernatants were obtained by growing *T*. *marneffei* in BHI broth at 37°C, 150 rpm for 7 and 14 days as described above. After harvesting, the supernatant was then concentrated using a 10 kDa cut-off Vivaspin concentrator (GE™ Healthcare). The protein concentrations were determined via Bradford assay [34] and 5 μg of the concentrated samples were loaded onto a 10% SDS-PAGE for Western blot analysis with MAb 4D1.

### Clinical urine specimens

Urine samples from HIV-seropositive patients with blood culture confirmed *T*. *marneffei* infections were collected between September 2004 to December 2009 (*n* = 66) at Maharaj Nakorn Chiang Mai Hospital, Chiang Mai, Thailand. The urine samples were obtained at the time of diagnosis and stored at −80°C until they were thawed on ice immediately prior to analysis. In some cases, urine samples were collected from patients diagnosed on clinical grounds and direct microscopic identification of fission yeasts from tissue specimens. As controls, urine samples from patients with other bacterial or fungal infections and from healthy individuals in our endemic area were also investigated (Table 1).

**Table 1:** Urine samples from patients infected with bacterial and fungal infections used in the study.

Clinical urine samples	Total
Culture confirmed of *T*. *marneffei*	66
Bacteria or yeast infections	42
Healthy from endemic area	70
Total	178

### Production of the ICT strip for the detection of *T*. *marneffei* cytoplasmic antigen

#### Preparation of MAb 4D1 conjugated colloidal gold nanoparticles and preparation of the conjugate releasing pads

The 60 nm particle of colloidal gold suspension, adjusted to pH 6.5 with 0.1 M Na_2_CO_3_, was divided into aliquots of 0.5 ml in microcentrifuge tubes. MAb 4D1 (100 μg/ml, 15 μl) in distilled water was added to each aliquot and allowed to react for 60 min. The residual surface of the gold particle was blocked by a 15 min treatment with 50 μg of 5% casein (w/v) dissolved in 2 mM Na_2_B_4_O_7_ pH 9.0 to give 0.5% casein final concentration. The conjugate was centrifuged at 10,000 rpm at 4°C for 15 min and the supernatant was discarded. The washing reagent of colloidal gold conjugate (0.5% w/v of casein, 20% w/v of saccharose dissolved in 2 mM Na_2_B_4_O_7_ pH 9.0) was added, and the conjugated gold particles were centrifuged at 10,000 rpm at 4°C for 15 min. After discarding the supernatant, the final volume of each aliquot was adjusted to 100 μl with colloidal gold conjugate washing reagent. To prepare the conjugate releasing pads, the antibody-gold conjugate suspension (0.5 μl) was applied to a piece of 3 x 3 mm glass fiber filter (Whatman Schleicher & Schuell, Dassel, Germany) and then baked in a 39°C incubator for 60 min. The conjugate releasing pads were further dried in a dehumidifier cabinet for an hour or overnight.

#### Immobilization of antigen and antibody onto analytical nitrocellulose membrane

Immobilizations of the antigen (TM CYA) and the antibody control (rabbit anti-mouse IgG, Sigma) onto nitrocellulose membrane were performed by the passive physical adsorption in the line pattern. The BioDot^®^7x100^TM^ (BioDot, Irvine, CA) dispensing platform was utilized for this purpose. The transferring of protein solution was performed by using a micro-syringe pump dispenser. The rate of transfer was 1 μl/cm. A 1.25 cm of nitrocellulose membrane (AE98 Fast; Whatman Schleicher & Schuell) was sprayed at the test line with TM CYA (> 50 kDa protein after diafiltration in 5 mM Tris-HCl pH 8.5) and rabbit anti-mouse IgG (1 mg/ml in 1x PBS pH 7.4) at the control line. The lined nitrocellulose membrane was immediately placed in a dehumidifier cabinet for 30 min. Subsequently, the non-lined surface of the nitrocellulose membrane was blocked by submersion in the blocking reagent (0.5% w/v of casein, 0.2% w/v of trehalose dissolved in 2 mM Na_2_B_4_O_7_ pH 9.0). The submersed nitrocellulose membranes were then placed on tissue papers and dried in a dehumidifier cabinet for 30 min. The blocked nitrocellulose membranes were stored at room temperature for 18–24 hr. before being assembled into the ICT strip system.

#### Construction and material composition of ICT strip

**Fig 1:**
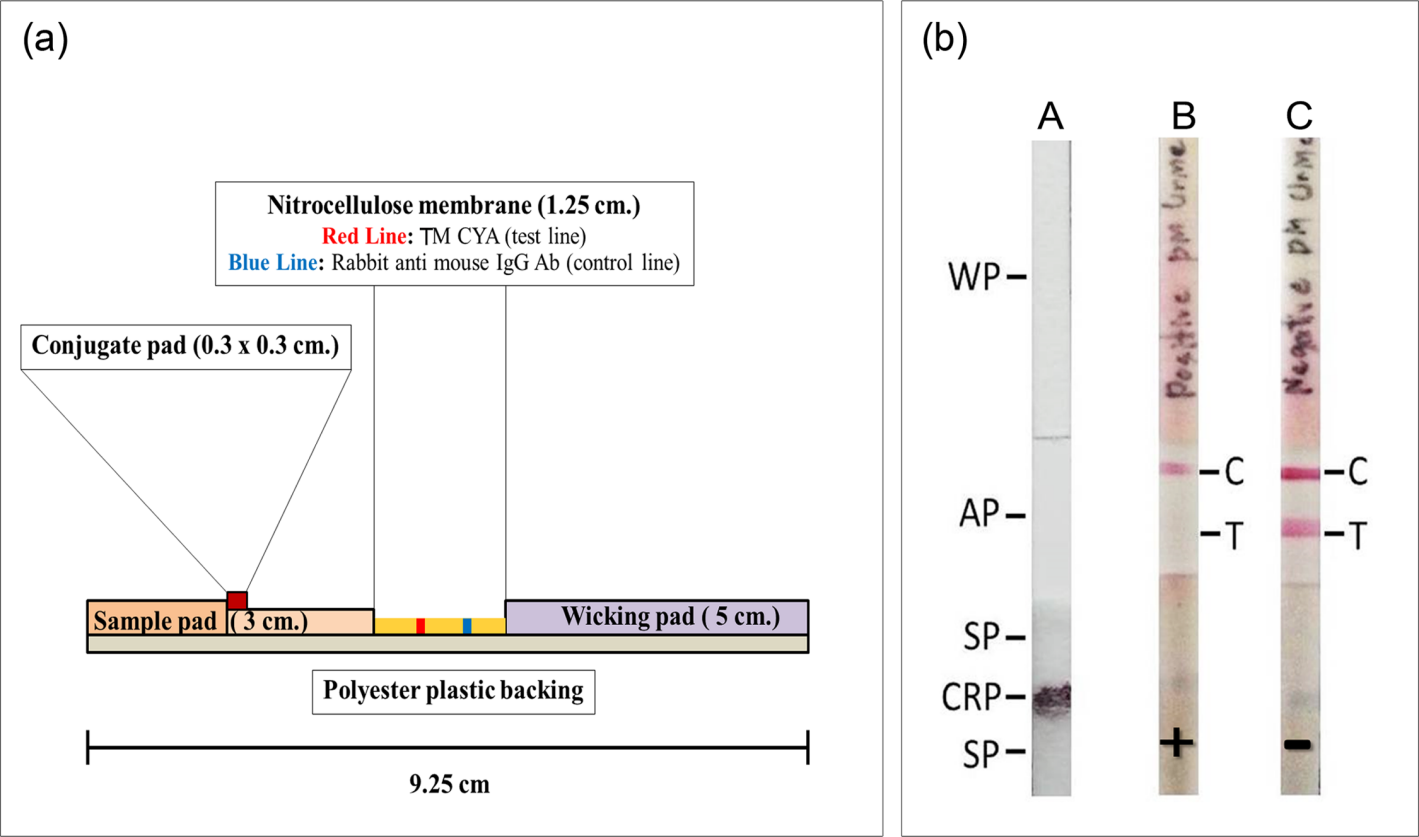
The ICT strip system of *T*. *marneffei*. (a) Cartoon of the components of the *T*. *marneffei* ICT strip. (b) A schematic diagram of the ICT strips for the detection of TM CYA antigen in urine. (A: Pre-run strip, B: Positive result, C: Negative result) WP, wicking pad; AP, analytical pad; SP, sample pad; CRP, conjugate releasing pad; C, control line; T, test line.

The ICT strip system was assembled utilizing 4 major components: the TM CYA and controlled antibody immobilized nitrocellulose membrane (Schleicher & Schuell), sample application pad (Glass fiber (GF33); Schleicher & Schuell), conjugate releasing pad (Glass fiber (GF33)) and wicking or absorbing pad (Chromatography paper, Whatman). They were manually assembled and held permanently in place with laminating plastic backing (Self-adhesive polyester backing, Schleicher & Schuell). The assembled cards were then cut into 3 mm wide strips using a BioDot^®^ CM 4000 R guillotine cutter. The detail of ICT strip components and strip format are demonstrated in Fig 1.

### Detection of *T*. *marneffei* antigen by the ICT strip

**Fig 2:**
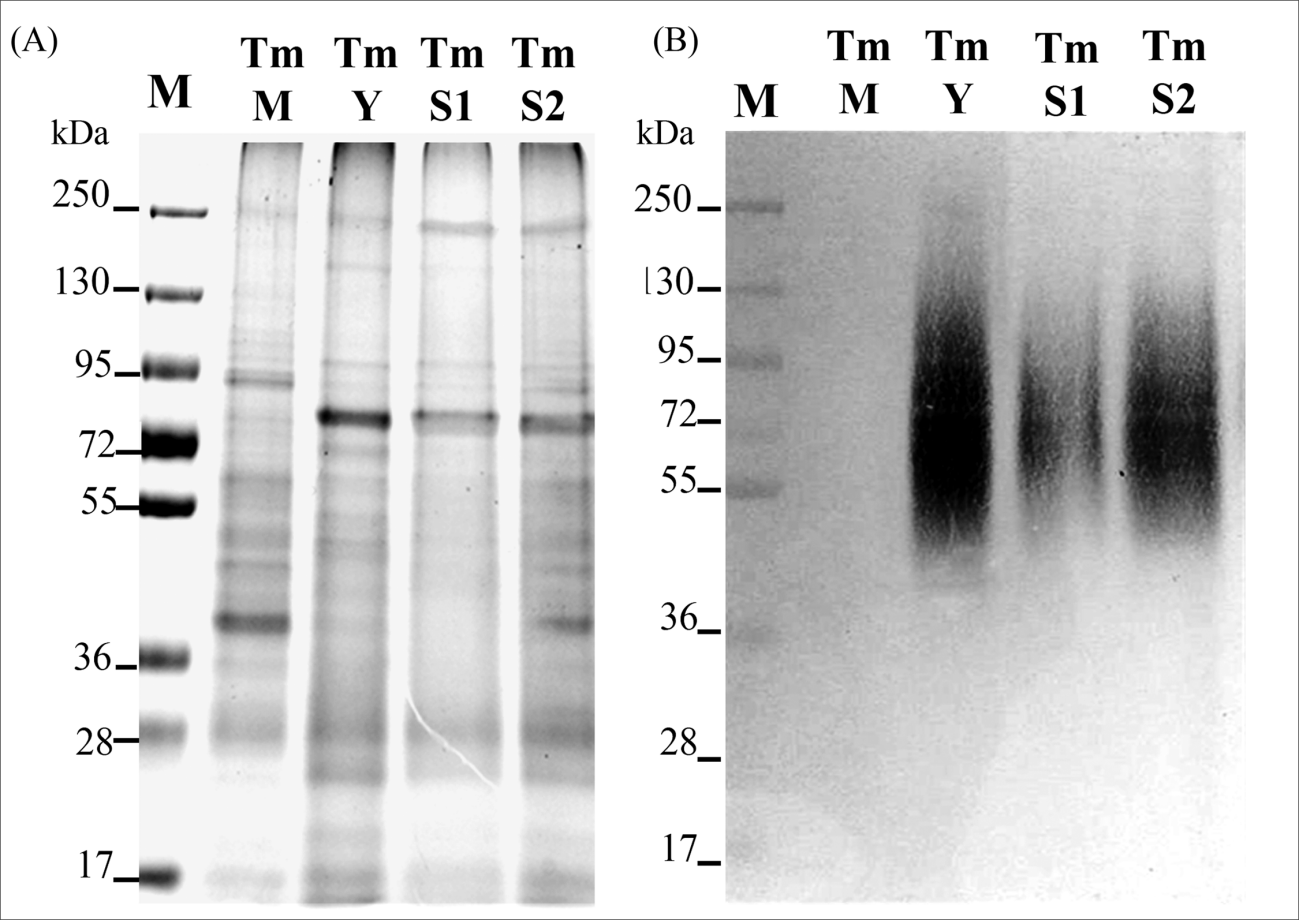
MAb 4D1 similarly labels prepared *T*. *marneffei* cytoplasmic yeast antigens and proteins released into *T*. *marneffei* culture supernatants. SDS-PAGE (A) and Western immunoblots (B) prepared with *T*. *marneffei* cytoplasmic mycelial antigen (TmM), yeast antigen (TmY) and the concentrated supernatants from day 7 (TmS1) and day 14 (TmS2) yeast cell cultures. MAb 4D1 was used in (B). The numbers on the left indicate relative molecular weights of markers (M).

The assembled ICT strip test was immersed vertically with the sample pad placed into 100 μl of specimen containing 2 μl of 0.25% Triton X 100 in 1xPBS pH 7.4, which results in the migration of the sample solution by capillary force through the conjugate releasing pad, rehydrating the colloidal gold conjugated MAb 4D1. As the solution moved across the test line, the colloidal gold conjugated MAb 4D1 would react with the immobilized TM CYA on the analytical nitrocellulose membrane generating a red color at the test line (Fig 2). Excess conjugate would migrate further to be captured by the rabbit anti-mouse IgG, generating a red color at the control line. In the absence of TM CYA in the sample, a negative outcome would be indicated by the appearance of reddish lines at both the control and test regions. In the presence of *T*. *marneffei* antigen in the sample, the *T*. *marneffei* antigen would bind to the limited amount of the MAb 4D1 and inhibit its reaction with the immobilized TM CYA on the test line, resulting in a single reddish line in the control region. With our system, the ICT was ready for reading by the naked eye within 20 minutes.

### Evaluation of the limit of detection (LOD) and cross reactivity of the ICT strips

The ICT strip test was used to detect various concentrations of TM CYA in urine samples. Urines from normal healthy controls were spiked with aliquots of TM CYA to give final TM CYA concentrations of 0.78, 1.56, 3.125, 6.25, 12.5, 25, 50 and 100 μg/ml. Then 100 μl of spiked and non-spiked urine solutions were transferred into the wells of 96 well “U” microtiter plates containing 2 μl of 0.25% Triton X 100 in 1xPBS pH 7.4. The ICT strips were then placed into the urines and the results were observed for the absence or presence of test lines after 20 min. The signal intensity of the test lines were also scanned by the UVP visionWorksLS analysis. The limit of detection of the test was set as the lowest concentration of TM CYA that gave a positive signal.

To study the specificity of the ICT, common pathogenic fungal proteins were spiked into urines from healthy controls. The fungal proteins were prepared as described in the preparation of TM CYA, and included cytoplasmic mold antigen (CMA) of *T*. *marneffei*, *C*. *albicans*, *C*. *neoformans*, *P*. *insidiosum*, *H*. *capsulatum*, *Penicillium* sp. and *A*. *fumigatus*. The urine samples individually spiked with 50 μg/ml of different fungal protein mixtures were then assayed as described above.

### Ethical statements

All clinical samples used in this study were received from an existing sample collection with specific permission. All samples were anonymized and the study was carried out in compliance with protocols approved by the Research Ethics Committee of Chiang Mai University, Chiang Mai, Thailand.

### Determination of fungal protein concentration

All fungal protein concentrations were determined by the dye binding method using bovine serum albumin (BSA; Sigma) as the standard [34].

## Results

### Characteristics of the MAb 4D1 monoclonal antibody and the TM CYA antigens

Western immunoblot analysis of cytoplasmic antigens from yeast and mycelial forms of *T*. *marneffei* as well as *C*. *albicans*, *C*. *neoformans*, *H*. *capsulatum*, *A*. *fumigatus*, *Sporothrix schenckii* and *Penicillium* sp. confirmed the specificity of MAb 4D1 for TM CYA (S1 Fig). The target antigenic mannoprotein of TM CYA recognized by MAb 4D1 has the molecular weight ranging from 50–150 kDa with the diffuse binding characteristics of “broad high molecular mass smear” (S1 Fig). These results correspond with the findings from the MAb 4D1 indirect ELISA, which similarly found that the MAb did not cross react with other pathogenic fungi [25].

In order to further confirm the reactivity of MAb 4D1 against secreted proteins of *T*. *marneffei*, yeast culture supernatants were examined by immunoblotting with MAb 4D1. Supernatants of *T*. *marneffei* cultivated for 7 or 14 days contained significant amounts of immunogenic components ranging from 50–150 kDa that were effectively recognized by MAb 4D1, and the reactivity was similar to the labeling of TM CYA (Fig 2).

### Development of an ICT for the detection of *T*. *marneffei* cytoplasmic yeast antigen

#### Limit of detection (LOD) of the ICT strip

**Fig 3:**
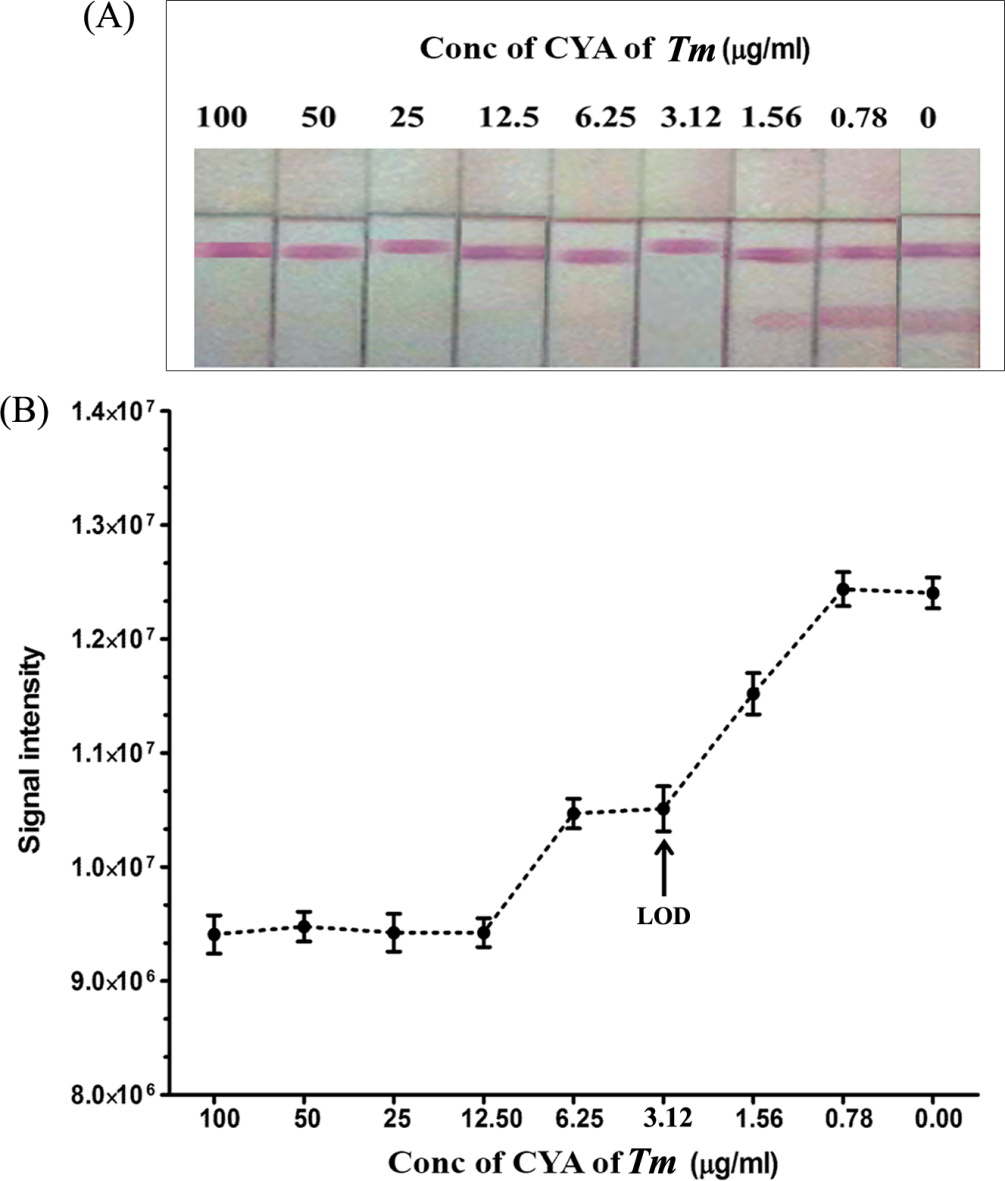
Determination of the limit of detection (LOD) of the ICT strip. (A) By visual inspection and (B) by UVP visionWorks LS scanner. Urine samples from healthy individuals were spiked with the indicated concentrations in μg/ml of TM CYA. NC, urine from healthy individuals as negative control; C: control line, T: test line.

The lower detection limit of the assay strip was determined with urine from normal controls spiked with TM CYA as described in Materials and Methods. The results showed that the visually observed LOD was at 3.125 μg/ml of TM CYA (Fig 3A). The LOD of this assay was also determined with the UVP visionWorks LS scanner, which gave the same result as that from the naked visualization. However, the scanner was equilibrated to the signal intensity of the negative control at 0.78 μg/ml of the TM CYA. Therefore, the LOD of this method may be as low as 3.125μg/ml when utilizing the color intensity reading (Fig 3B).

#### Specificity study of the ICT strip

**Fig 4:**
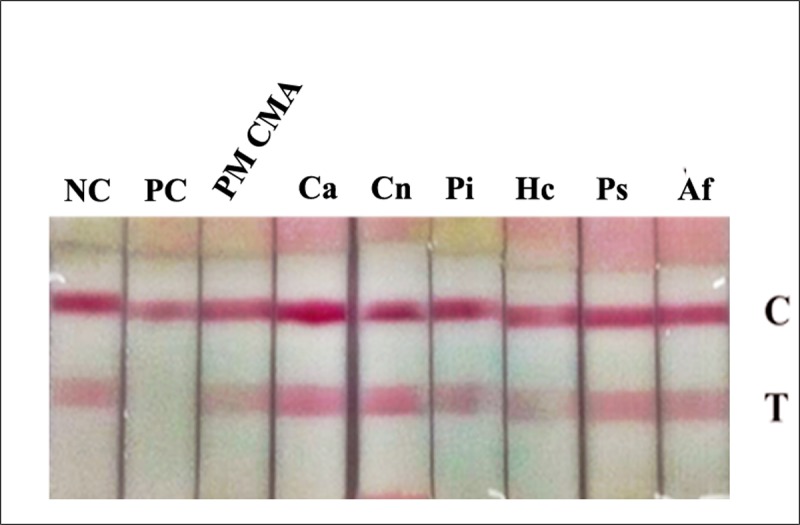
The ICT system does not detect antigens from other common fungal pathogens. Urine samples from healthy volunteers were spiked with TM CYA (PC), TM CMA, *C*. *albicans* (Ca), *C*. *neoformans* (Cn), *P*. *insidiosum* (Pi), *H*. *capsulatum* (Hc), *Penicillium* sp. (Ps), *A*. *fumigatus* (Af) at 50 μg/ml each. NC, normal urine negative control; C, control line; T: test line.

To evaluate the specificity of the ICT, TM CMA as well as proteins isolated from common fungal pathogens, including *C*. *albicans*, *C*. *neoformans*, *P*. *insidiosum*, *H*. *capsulatum*, *Penicillium sp*. and *A*. *fumigatus* was spiked into urine from healthy volunteers at a concentration of 50μg/ml. The results showed that the ICT assay was highly specific for detecting only the yeast phase antigen of *T*. *marneffei*. No cross-reactivity was observed (Fig 4).

#### Detection of *T*. *marneffei* antigen in urine specimens by the ICT strip

The ICT was used to test urine samples of patients with hemoculture confirmed *T*. *marneffei* infection. Out of the 66 patients with *T*. *marneffei* infection, 58 urine samples gave positive test results and 8 were negative (S2 Fig). In addition, 3 of 8 urine samples showing false negative were collected from talaromycosis marneffei patients after treatment with anti fungal agent and the antigenuria may be lower than 3.25 μg/ml. There were no positive tests with the 42 urine samples from patients with bacterial or other fungal diseases or with the 70 samples of normal healthy volunteers (S3 and S4 Figs). Thus, these results show that the ICT exhibited a diagnostic sensitivity, specificity and accuracy of 87.87%, 100% and 95.5%, respectively.

## Discussions

Laboratory diagnosis of talaromycosis marneffei requires accurate and rapid diagnosis to facilitate the initiation of appropriate treatment. The microbiological culture of *T*. *marneffei* from a clinical specimen is the gold standard method for diagnosis of talaromycosis marneffei, but it is not suitable for early diagnosis because it takes at least 7 to 10 days. Since antigen loads are frequently high and antibody responses may be muted in immunocompromised patients, antigen detection is a promising diagnostic approach, especially in areas of endemicity [9].

The present study demonstrates the successful development of a novel ICT system for rapidly diagnosing *T*. *marneffei* infection. The system employs the murine IgG1 MAb 4D1, which is highly specific against an immunogenic mannoprotein on an N-linked glycoprotein [26]. MAb 4D1 reacts with both the cytoplasmic and secreted yeast antigen of *T*. *marneffei* cultured in BHI broth. Although CYA of *T*. *marneffei* was used as an immunogen for BALB/c mice to produce MAb 4D1, components of CYA also secreted into culture broth. This observation suggested that the secreted antigens of *T*. *marneffei* found in blood, urine and possibly other secretions during infection could be detected by MAb 4D1. The results of the present study support this contention. Specifically, tests by immunoblotting show that MAb 4D1 reacts strongly with yeast phase antigen of *T*. *marneffei*, which is similar to our prior results with an indirect ELISA using MAb 4D1 [27]. MAb 4D1 doesn’t react with *T*. *marneffei* mycelia antigens or cytoplasmic antigens of *C*. *albicans*, *C*. *neoformans*, *H*. *capsulatum*, *P*. *insidiosum*, *A*. *fumigatus* or *Penicillium* sp. Moreover, the target antigenic mannoprotein of MAb 4D1 appears to be abundantly expressed and is highly conserved in most isolates of *T*. *marneffei* [27]. Therefore, this glycoprotein antigen was a potential target identifiable by MAb 4D1 for the diagnosis of *T*. *marneffei* infection.

Our recent publication of the MAb 4D1 inhibition ELISA using serum demonstrated the utility of harnessing this MAb for diagnosis [27]. The ELISA detected antigenemia in all 45 patients with hemoculture confirmed *T*. *marneffei* infection, resulting in a 100% diagnostic sensitivity (45/45). The assay revealed that the mean antigen concentration of these patients was 4.32 μg/ml, and serum with an antigen level ≥ 0.07 μg/ml was considered to be positive. In addition to being useful for diagnosis, the inhibition ELISA could be used to monitor the success of therapy. However, the assay was found to be time consuming, especially as it required specialized equipment and skilled personnel to perform the testing.

The diagnostic performances of the present ICT were determined in urine samples from culture confirmed talaromycosis marneffei. The sensitivity, specificity and accuracy of the assay were 87.87% (58/66), 100% (112/112) and 95.5% (170/178), respectively. Although all urine samples in this study were collected from patients with a positive blood culture of *T*. *marneffei*, the sensitivity of the ICT was 87.87%. Indeed, 76.7% of blood cultures from talaromycosis patients were positive [11], thus the sensitivity of the ICT should be lower in the case of urine samples from patients with negative blood cultures for *T*. *marneffei*. For specificity, no cross reactivity of this assay was observed when using clinical urine samples from patients with other fungal and bacterial infections.

The limit of detection (LOD) for the quantitative detection was 3.125μg/ml by both a visual method and by measurements using a UVP visionWorksLS scanner. However, the signal intensity from the scanner was equilibrated to the signal intensity of the negative control at 0.78 μg/ml of the TM CYA inhibitor. So, the LOD of this method may be lower than 3.125μg/ml when utilizing color intensity reading.

The diagnostic performance of the ICT was in agreement with previous *T*. *marneffei* antigenuria detection methods such as EIA mediated rabbit anti-whole arthroconidia of *T*. *marneffei* IgG-FITC conjugate and MAb based sandwich ELISA [15,35]. The diagnostic sensitivities of these assays were 97% and 72% respectively.

In order to detect antigen in clinical samples, urine is a highly desirable specimen in resource-limited settings, but antigenemia is more reflective directly in prognosis of disease. Unfortunately, our ICT is subject to a significant limitation for the detection *T*. *marneffei* antigenemia due to the detachment of colloidal gold particles from the strip in the presence of serum (data not shown).

In conclusion, the ICT test developed in this study exhibited high specificity and sensitivity for *T*. *marneffei* antigen present in clinical urine samples of patients with talaromycosis marneffei. This 20 minute assay is remarkably easy to perform and does not require specialized equipment or skilled personnel. Furthermore, the ICT strips are stable for about 2 years at room temperature. We suggest that this ICT should considered for clinical application for the rapid diagnosis of *T*. *marneffei* infection and, consequently, the proper management and treatment of the disease.

## Supporting information

S1 Fig **Reactivity of anti-yeast specific MAb 4D1** against *T*. *marneffei* antigens (Tm) in both yeast (CYA) and mycelial forms (CMA), and antigens from common fungal pathogens including *Histoplasma capsulatum* (HC), *Sporothrix schenckii* (SS), *Candida albicans* (CA), *Penicillium* spp. (PS), *Cryptococcus neoformans* (CN), *Pseuallescheria boydii* (PB), and *Aspergillus fumigatus* (AF) by SDS-PAGE (A) and Western blotting analysis (B).
(TIF)

S2 Fig Representative ICT strips subjected to urine samples from patients with hemoculture confirmed *T*. *marneffei* (N = 30 of 66 total of *T*. *marneffei* infection). NC: negative control; C: control line; T: test line; PC: positive control.
(TIF)

S3 Fig Representative ICT strips subjected to urine samples from patients with the bacterial and other fungal infections (N = 20 of 42 total bacterial and other fungal infections). NC: negative control, PC: positive control; C: control line; T: test line.
(TIF)

S4 Fig Representative ICT strips subjected to urine samples from normal healthy persons living in *T*. *marneffei* endemic area (N = 22 of 70 total healthy controls). NC: negative control, PC: positive control; C: control line; T: test line.
(TIF)
