# Supplementary figures and images for: Development and characterization of an immunochromatographic test for the rapid diagnosis of Talaromyces (Penicillium) marneffei
Source: PLoS One. 2018 Apr 11;13(4):e0195596. doi: 10.1371/journal.pone.0195596 (PMC5895032; doi:10.1371/journal.pone.0195596)

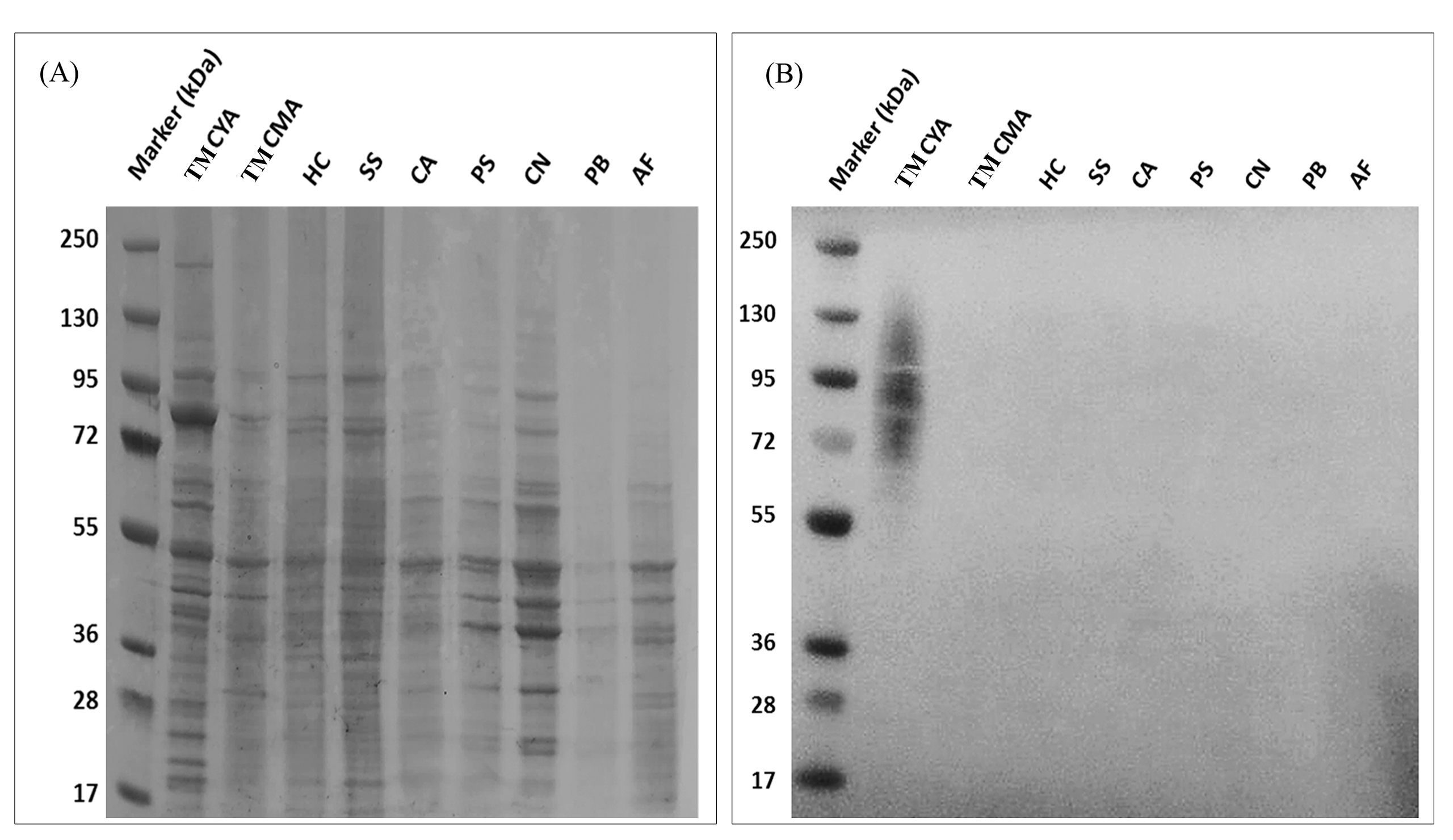

Supplement: S1 Fig — Reactivity of anti-yeast specific MAb 4D1 against T. marneffei antigens (Tm) in both yeast (CYA) and mycelial forms (CMA), and antigens from common fungal pathogens including Histoplasma capsulatum (HC), Sporothrix schenckii (SS), Candida albicans (CA), Penicillium spp. (PS), Cryptococcus neoformans (CN), Pseuallescheria boydii (PB), and Aspergillus fumigatus (AF) by SDS-PAGE (A) and Western blotting analysis (B). (TIF) [file pone.0195596.s001.tif]

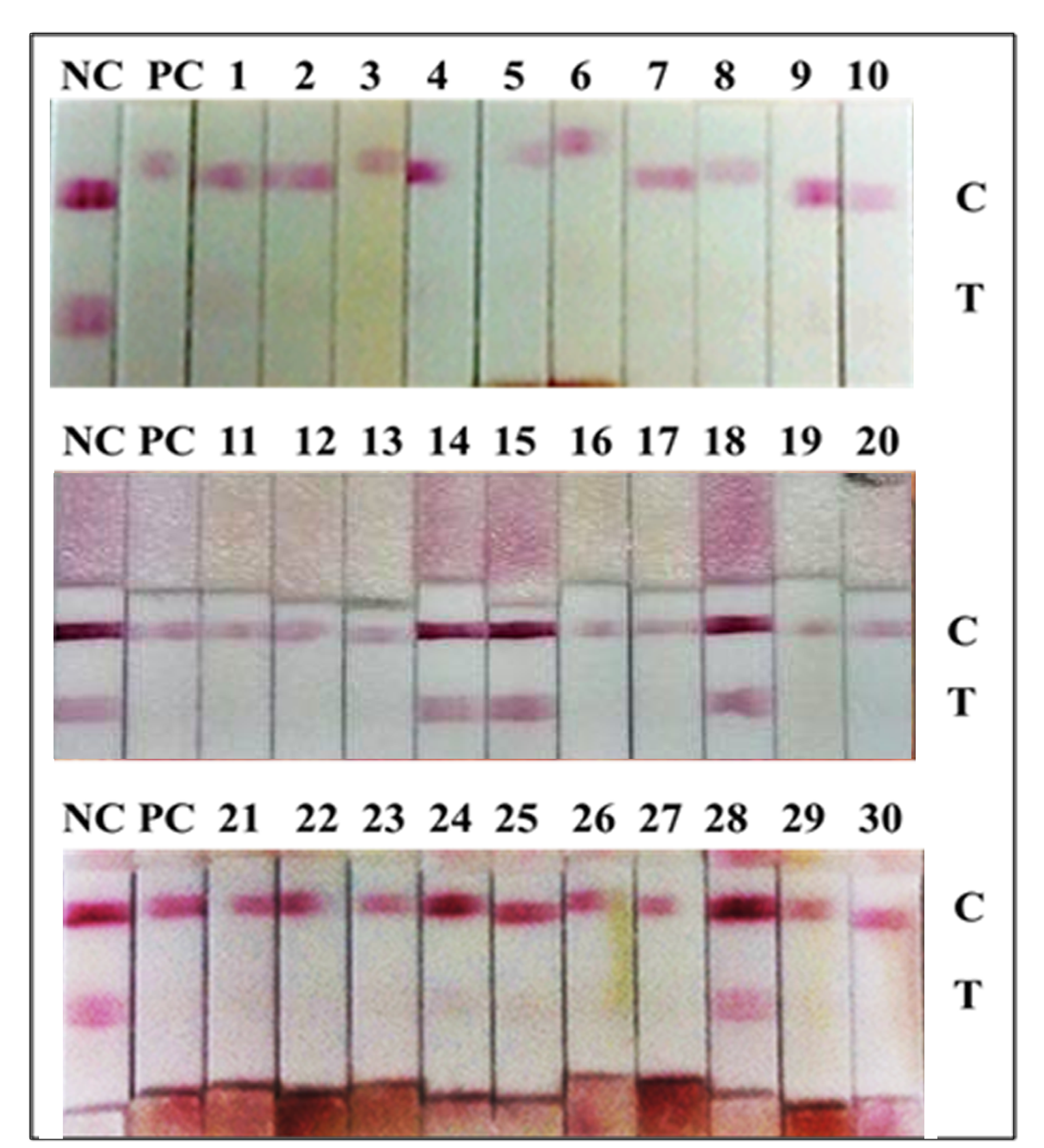

Supplement: S2 Fig — NC: negative control; C: control line; T: test line; PC: positive control. (TIF) [file pone.0195596.s002.tif]

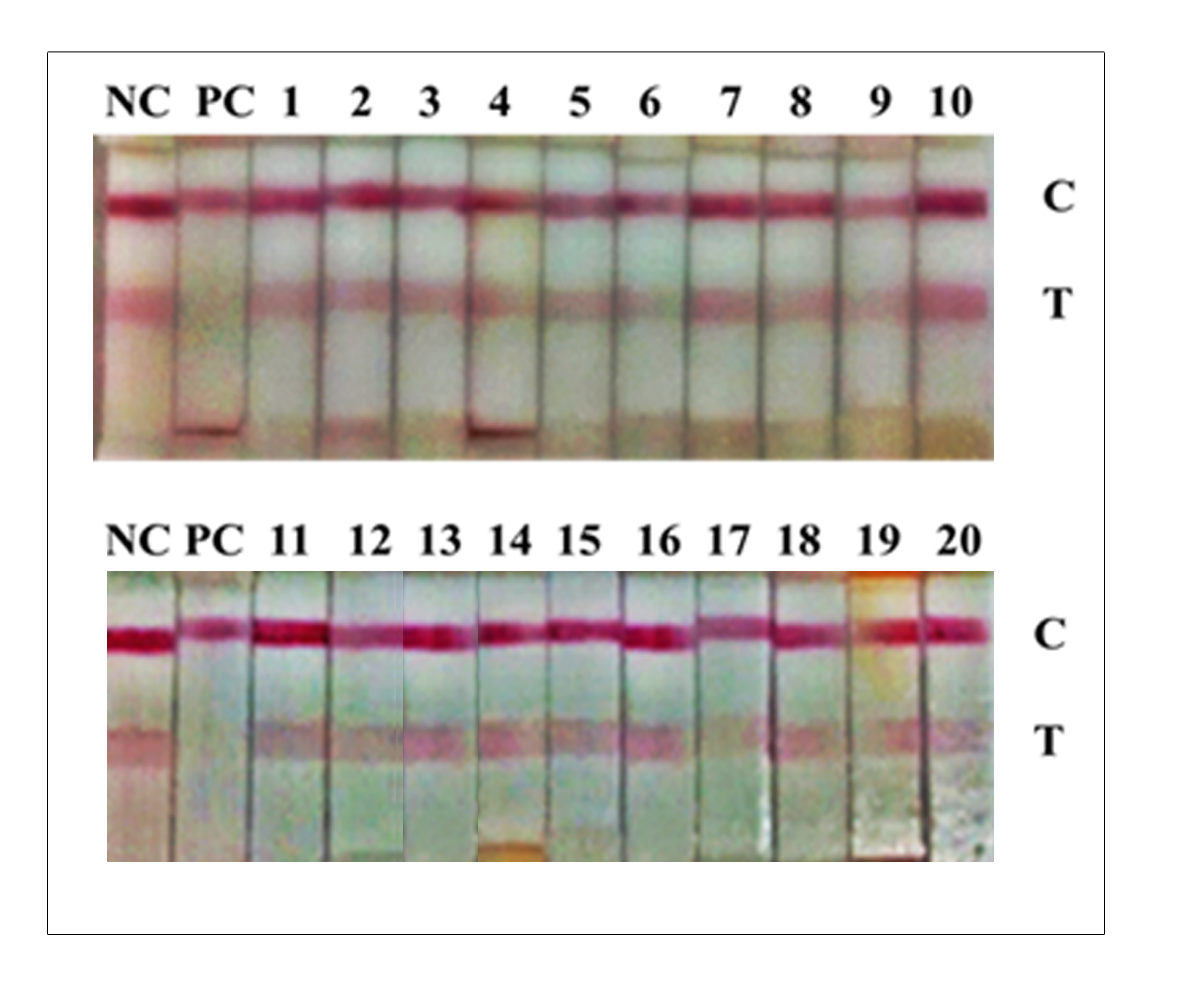

Supplement: S3 Fig — NC: negative control, PC: positive control; C: control line; T: test line. (TIF) [file pone.0195596.s003.tif]

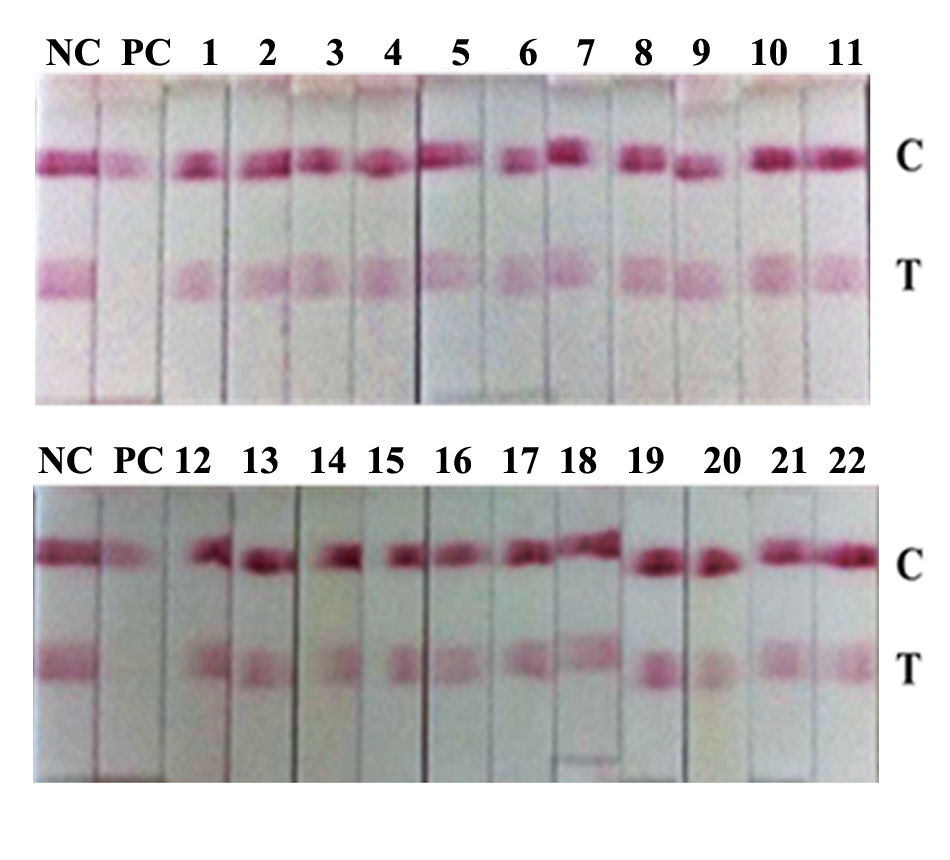

Supplement: S4 Fig — NC: negative control, PC: positive control; C: control line; T: test line. (TIF) [file pone.0195596.s004.tif]
